# Optical coherence tomography angiography as a tool for diagnosis and monitoring of sickle cell related eye disease: a systematic review and meta-analysis

**DOI:** 10.1038/s41433-025-03814-1

**Published:** 2025-05-22

**Authors:** Kirsty Clarke, Ankith Mannath, Marco Anastasi, Mohamed Nasr, Shengning Pan, Konstantinos Balaskas, Christiana Dinah, Marinko V. Sarunic, Riaz Asaria

**Affiliations:** 1 Royal Free London NHS Foundation Trust, London, UK; 2 University College London Medical School, Faculty of Medical Science, London, UK; 3 University College London Department of Statistical Science, London, UK; 4 NIHR Moorfields Biomedical Research Centre, London, UK; 5 Institute of Ophthalmology, University College London, London, UK; 6 Moorfields Eye Hospital NHS Foundation Trust, London, UK; 7 London North West University Healthcare NHS Trust, London, UK; 8 Department of Medical Physics and Biomedical Engineering, University College London, London, UK

**Keywords:** Retinal diseases, Medical imaging

## Abstract

Sickle cell retinopathy (SCR) is an ocular manifestation of sickle cell disease (SCD). In SCR abnormal sickling of erythrocytes is associated with sight-threatening complications such as neovascularisation, vitreous haemorrhage, maculopathy and retinal detachment. Optical coherence tomography angiography (OCTA) is a novel imaging modality enabling non-invasive assessment of retinal vasculature. This systematic review provides an up-to-date evaluation of the role of OCTA in SCR diagnosis and management. We searched MEDLINE/PubMed, Cumulated Index to Nursing and Allied Health Literature (CINAHL), SCOPUS and Cochrane Central Register of Controlled Trials (CENTRAL) electronic databases. The methodological quality of included studies was evaluated according to the STrengthening the Reporting of OBservational studies in Epidemiology (STROBE) recommendations. 31 studies met the inclusion criteria, and 26 suitably complied with the STROBE recommendations. Participant characteristics, including haemoglobin genotype, Goldberg staging, and visual acuity, were reported in twenty-eight (93%), twenty-six (86.6%), and fifteen (50%) studies, respectively. There was no consistent association between OCTA findings and haemoglobin genotype, Goldberg staging or visual acuity. Meta-analysis revealed that foveal avascular zone (FAZ) size and mean vessel density of the superficial and deep capillary plexi were significantly altered in patients with SCR compared to healthy controls. The mean vessel density of the superficial and deep capillary plexus was significantly lower in patients with SCR. OCTA can quantitatively detect retinal vascular remodelling in patients with SCR. Further research should focus on the clinical utility of OCTA for predicting SCR progression and its role in automating SCR staging using machine learning techniques.

## Introduction

Sickle cell disease is the most common inherited disease worldwide [1]. It arises from a range of pathogenic variants of genes responsible for encoding haemoglobin, encompassing HbSS, HbSC, and HbS beta-thalassaemia genotypes. Clinical manifestations of sickle cell disease occur when deoxygenated erythrocytes deform into a sickle shape, resulting in intermittent vaso-occlusion of microcirculatory networks and multi-organ dysfunction [2]. In the retina, intermittent vaso-occlusive events result in characteristic findings called sickle cell retinopathy (SCR).

SCR has long been considered a disease of the peripheral retina. The Goldberg classification recognises peripheral retinal changes, such as arterial occlusions, arteriovenous anastomoses (hairpin loops) and neovascularisation (sea fan) as early indicators of SCR (Stages I-III). Such changes precede the onset of sight-threatening complications, including vitreous haemorrhage (stage IV) and retinal detachment (stage V) [3, 4]. Based on this classification, a combination of clinical examination, optical coherence tomography (OCT), and fluorescein angiography (FA) remain the current gold standard for SCR diagnosis and monitoring [5]. However, macular microangiopathy is increasingly recognised as an early biomarker for SCR that could be detected earlier than peripheral retinal changes [6, 7].

Optical coherence tomography angiography (OCTA) is a relatively new, non-invasive imaging modality that does not require the intravenous injection of contrast agents to achieve detailed quantitative representations of the macula’s vasculature [8, 9]. Several characteristics of OCTA make it especially suitable for investigating SCR. In individuals with symptomatic retinal changes, OCTA reliably identifies macular microangiopathy that may be less discernible using FA [9]. Furthermore, evidence suggests OCTA could be more sensitive than existing techniques for detecting asymptomatic retinovascular remodelling, providing biomarkers for SCR screening [10].

Many quantitative OCTA metrics, including foveal avascular zone (FAZ) size, microvascular density, and vessel tortuosity, have already been successfully mapped against existing systems for SCR grading, enabling early refinement of SCR screening protocols [10]. Moreover, Zhou et al. have explored sequential OCTA imaging and developed the concept of an intermittent perfusion index [11]. This index reflects the systemic disease burden and can assess the response to systemic therapy. OCTA outputs like these facilitate large-scale data analysis and support the development of AI algorithms. Such advancements could prove pivotal in elucidating the relationship between SCR and systematic disease outcomes such as silent infarcts, serological changes and response to systemic therapeutics [12].

In the context of SCR, OCTA offers a safe, cost-effective and future-proof solution for monitoring what can be an unpredictable disease that is otherwise difficult to prognosticate. The quantitative metrics produced by OCTA enable early and objective detection of sickle cell maculopathy and could be a surrogate marker for disease progression in the peripheral retina or even the microvasculature elsewhere in the body [12]. With the growing interest in OCTA as a tool to diagnose and monitor SCR, previous attempts to synthesise evidence for the role of OCTA in SCR have become outdated [13]. We aim to systematically search the literature, providing a current evaluation of the role of OCTA in SCR diagnosis and monitoring.

## Materials (subjects) and methods

### Search strategy

An information specialist from University College London was consulted to develop our search strategy. We searched the MEDLINE/PubMed, Cumulated Index to Nursing and Allied Health Literature (CINAHL), SCOPUS and Cochrane Central Register of Controlled Trials (CENTRAL) electronic databases using a combination of descriptors, including Medical Subject Headings and Descriptors in Health Sciences terms, as well as contractors of descriptors.

The systematic review was not restricted to English publications; studies written in any language were included. The Preferred Reporting Items for Systematic Reviews and Meta-Analyses (PRISMA) document was used as a guide for the systematic review. Terms used for the search were related to the studies population (“sickle cell” OR “sickle cell disease” OR “sickle cell anaemia” OR “sickle cell anaemia” OR “sickle cell retinopathy” OR “sickle” OR “sickle cell maculopathy”) and to the intervention performed (“optical coherence tomography angiography” OR “optical coherence tomography angiography OCTA” OR “OCT A” OR “OCT angiography” OR “OCTA”). Finally, references were found by “snowballing”, where references of included articles were manually reviewed to identify additional studies meeting the review inclusion criteria.

### Inclusion and exclusion criteria

The inclusion criteria for the review, as instructed by the “PICO” framework [14], are listed below.

### Inclusion criteria

- Any language
- 2010-present
- Reported data includes patients with sickle cell disease
- Report OCTA outcomes
- Study type: randomised clinical trials, clinical trials, observational studies, 128 case reports, case series, abstracts published in scientific journals, case reports, 129 letters to the editor, and publications in conference proceedings.

### Exclusion criteria

- No data for patients with sickle cell disease.
- No qualitative or quantitative OCTA data was reported.

### Study identification and selection

Two independent authors (KC, MA) read the titles and abstracts of each pre-selected paper to identify studies that met the inclusion criteria. Both authors were blinded to one another’s opinion, achieving an agreement of 90.6%. The full-text articles were then read by two additional authors (RA, MN), who made the final selection, ensuring that the systematic review criteria were met. Any differences between the authors were resolved through discussion and dialogue, and in the case of persistent disagreement, a senior professor made the final decision (RA).

### Data extraction

Two authors (KC, MA) collected the data using a bespoke collection form designed to correspond to the data extraction described by Guerra et al. in their 2019 systematic review of OCTA to detect changes in sickle cell retinopathy [13]. A third independent author (MN) reviewed the extracted data. The study characteristics extracted included publication date, geographical origin, title, study design, presence of a control group, other interventions performed, supervision, and financing.

### Analysis of study quality

The methodological quality of the studies included in this review was evaluated according to the STrengthening the Reporting of OBservational studies in Epidemiology (STROBE) recommendations [15].

### Analysis of standardisation of results

The data collected were standardised, and all articles were considered to summarise:

- Association between OCTA image analysis findings and study population characteristics.
- Qualitative analysis of OCTA features for patients with SCR and their respective frequencies compared to controls.
- Quantitative analysis of OCTA images in patients with sickle cell retinopathy compared to controls.

Statistical support for the project was provided by the Biostatistics Group at the Joint Research Office at University College London. SPSS statistics Software IBM®Version 27 was used for data management (SP). Meta-analysis and plots were coded using R package Meta.

## Results

### Identification and selection of studies

**Fig. 1:**
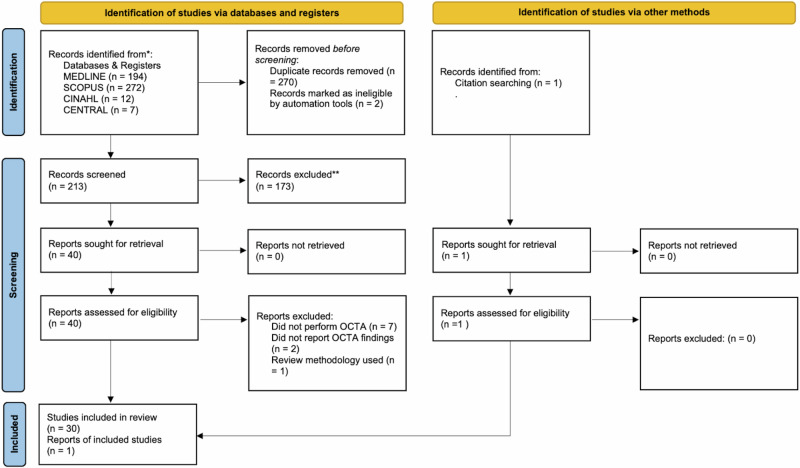
PRISMA flow diagram of study selection process. This flowchart illustrates the systematic process used to identify, screen, assess, and include studies in the review. A total of 485 records were initially identified from database searches (MEDLINE, SCOPUS, CINAHL, and CENTRAL), with an additional record from citation searching. After removal of duplicates and ineligible records, 213 were screened. Following exclusion of 173 studies, 40 full-text reports were assessed for eligibility. Of these, 10 were excluded due to not performing or reporting OCTA, or using review methodology. Ultimately, 30 studies and 1 additional report were included in the final synthesis. Symbols: solid arrows indicate progression through screening stages.

The PRISMA flow diagram showing the study selection process is shown in Fig. 1. Of the 485 references obtained using the search strategy, 272 remained once duplicates were removed. Of the 272 studies, 40 complete texts were selected for data extraction and full-text review. Of these, ten studies were excluded: seven because the wrong imaging was conducted, one because there was inadequate reporting of OCTA results, one because the wrong outcome was evaluated and one because it was a review article. One study was identified by manually reviewing citations of included studies. Finally, 31 studies met the inclusion criteria for this systematic review (Fig. 1) [1, 6, 8–12, 16–36].

### Characteristics of selected studies

The main characteristics of the studies included in the systematic review are reported in Table 1.

**Table 1:** Summary of study characteristics.

Reference	Country	Publication Year	Sample size eyes	Mean age	Study design	Control group	OCTA device	Technology	Scan
Mgboji et al.	USA	2022	2 (4)	32	Case Series (Prospective)	No	Optovue RTVue XR (Optovue Inc, Fremont, California, USA)	SD-OCT	3 × 3 mm, 6 × 6 mm
Minvielle et al.	France	2016	9 (18)	41	Cross-sectional (Retrospective)	Yes	AngioVue, RTVue XR, Avanti, Optovue Inc,Fremont, California, USA	SD-OCT	3 × 3 mm
Pahl et al.	USA	2017	16 (32)	15	Cross-sectional (Prospective)	Yes	Cirrus HD- OCT 5000 with Angioplex OCT Angiography	SD-OCT	3 × 3 mm; 6 × 6 mm; 8 × 8 mm
Fares et al.	France	2021	78 (151)	37	Cross-sectional (Retrospective)	Yes	Spectralis HRAþOCT (Heidelberg Engineering, Heidelberg, Germany)	SD-OCT	30 × 25
Mokrane et al.	France	2020	24 (46)	34	Cohort (Prospective)	Yes	Triton Plus, Topcon, Tokyo, Japan	SS-OCT	3 × 3 mm
Han et al. [33]	USA	2017	46 (82)	36	Cross-sectional (Prospective)	No	Optovue RTVue XR (Optovue Inc, Fremont, California, USA)	SD-OCT	3 × 3 mm, 6 × 6 mm
Cano et al.	USA	2020	12 (24)	40	Cohort (Prospective)	Yes	Optovue, Inc., Fremont, CA	SD-OCT	6 × 6 mm
Croisé et al.	France	2020	31 (56)	31	Case Series (Retrospective)	No	Topcon DRI OCT Triton model	SS-OCT	3 × 3 mm, 6 × 6 mm
Falavarjani et al.	USA	2016	10 (18)	37	Cross-sectional (Retrospective)	No	Optovue RTVue XR (Optovue Inc, Fremont, California, USA)	SD-OCT	3 × 3 mm
Lynch et al.	USA	2020	52 (52)	33	Cross-sectional (Retrospective)	Yes	Avanti RTVue-XR; Optovue, Fremont, CA, USA	SD-OCT	3 × 3 mm
Zhou et al.	USA	2020	31	31	Cross-sectional (Retrospective)	Yes	Avanti RTVue-XR; Optovue, Fremont, CA, USA; Angioanalytics™, version 2018.0.0.16	SD-OCT	3 × 3 mm
Jung et al.	USA	2018	2 (2)	34	Case Report	No	OCTA, Investigational, prototype, Carl Zeiss Meditec, Dublin, CA Carl Zeiss Meditec, Dublin, CA	SS-OCT	12 ×12 mm
Sambhav et al.	USA	2017	3 (3)	19	Case Report	No	Optovue RTVue XR (Optovue Inc, Fremont, California, USA)	SD-OCT	8 × 8 mm
Martin et al.	France	2017	3 (06)	Unknown	Case Series	No	Optovue RTVue XR (Optovue Inc, Fremont, California, USA),	SD-OCT	6 × 6 mm
Grover et al.	USA	2016	1 (1)	19	Case Report	No	Optovue, RTVue XR, Avanti, Optovue,Inc, Fremont, California, USA	SD-OCT	8 × 8 mm
Monteiro et al.	Portugal	2022	16 (32)	16	Case Series (Retrospective)	No	Spectralis OCTA® (Heidelberg Engineering)	SD-OCT	HR 30°x15°
Alam et al.	USA	2017	20 (37)	40	Cross-sectional (Retrospective)	Yes	Optovue, Fremont, CA, USA	SD-OCT	6 × 6 mm
Khansari et al.	USA	2017	17 (34)	42	Cohort (Prospective)	Yes	Optovue Inc, Fremont, California, USA	SD-OCT	6 × 6 mm, 3 × 3 mm
Alam 2019 [22]	USA	2021	48 (85)	43	Case series (Prospective)	Yes	Angiovue SD-OCT angiography system (Optovue, Fremont, CA)	SD-OCT	6 × 6 mm
Alam et al. [10]	USA	2017	18 (32)	40	Cross-sectional (Prospective)	Yes	AngioVue (OPTOVUE, Fremont, CA, USA)	SD-OCT	3 × 3 mm
Zhou et al. [11]	USA	2021	13	31	Cross-sectional (Retrospective)	Yes	Avanti RTVue-XR; AngioAnalytics software version 2017.1.0; Optovue, Fremont, CA, USA	SD-OCT	3 × 3 mm
Pinhas et al.	USA	2022	11 (11)	33	Case series (Prospective)	Yes	Avanti RTVue-XR; AngioAnalytics software version 2017.1.0; Optovue, Fremont, CA, USA	SD-OCT	3 × 3 mm
Bistour et al.	France	2023	28 (42)	Unknown	Cross-sectional (Retrospective)	No	(Carl Zeiss Meditec Inc., USA)	SS-OCT	12 ×12 mm
Han et al. [6]	USA	2018	19 (36)	31	Cross-sectional (Prospective)	No	Optovue RTVue XR (Optovue Inc, Fremont, California, USA),	SD-OCT	3 × 3 mm
Han et al. [19]	USA	2015	05 (10)	38	Cross-sectional (Prospective)	No	Optovue RTVue XR (Optovue Inc, Fremont, California, USA),	SD-OCT	Unknown
Sanfilippo et al.	USA	2015	1 (2)	33	Case Report	No	AngioVue, Inc, Fremont, California, USA RTVue XR Avanti, Optovue	SD-OCT	Unknown
Ong et al.	USA	2020	34 (68)	17	Case series (Prospective)	Yes	Optovue RTVue XR Avanti; Optovue Inc, Fremont, California, USA	SD-OCT	3 × 3 mm, 6 × 6 mm
Abdelkader et al.	Egypt	2022	15 (30)	11	Cross-sectional (Retrospective)	No	RS-3000 OCT Retina Scan Advance 2, Nidek Co., Japan	SOLO-OCT	3 × 3 mm, 6 × 6 mm
Grego et al.	Italy	2020	18 (36)	11	Cross-Sectional (Prospective)	No	Topcon Triton, Tokyo Optical Co.	SS-OCT	3 × 3 mm
Roemer et al.	Switzerland	2019	10 (19)	8	Case series (Retrospective)	Yes	AngioVue Imaging System; Optovue,	SD-OCT	3 × 3 mm

### Assessment of study quality

Compliance with the STROBE recommendations for observational studies was evaluated, and all studies, except for five case reports, were suitably compliant. The completed checklist is available in the supplementary materials. The GRADE criteria was also used to assess study quality and a summary of the results can be found in the supplementary materials. All but four studies were found to have low risk of bias in domains of “Inconsistency” and “Publication Bias”. However, either a moderate or high risk in domains of “Risk of Bias”, “Indirectness” and “imprecision” was reported for all the studies, indicating results must be interpreted with caution.

### Qualitative features of sickle cell disease on OCTA

The main qualitative findings in patients with SCD represented as frequencies are reported in Table 2. OCTA Imaging findings are categorised by OCTA technologies scan area size (standard, widefield, ulta-widefield) and were as follows: the presence of areas of nonperfusion in the superficial capillary plexus (SCP) and deep capillary plexus (DCP), irregularity of the FAZ, increased FAZ area, increased vascular tortuosity, increased vascular diameter and disruption of the perifoveal anastomotic capillary arcade. The presence of nonperfusion areas in the SCP or DCP was the most frequent change, and the DCP nonperfusion area was the finding described in the largest number of studies.

**Table 2:** Summary of Qualitative OCTA Features.

OCTA finding	Reference	Sample (eyes)	Frequency n (%)
*Non-flow area SCP*	Standard OCTA Imaging		
	Pahl et al.	30	6 (20)
	Falavarjani et al.	18	10 (55)
	Minvielle et al.	18	13 (72)
	Sambhav et al.	3	3 (100)
	Grover et al.	1	1 (100)
	Mokrane et al.	46	2 (4)
	Mgboji et al.	4	4 (100)
	Han et al. [33]	82	15 (38)
	Pinhas et al.	11	3 (27)
	Han et al. [19]	10	2 (20)
	Sanfilippo et al.	2	1 (50)
	Ong et al.	68	27 (40)
	Abdelkader et al.	6	4 (66)
	Grego et al.	36	6 (17)
	Widefield OCTA Imaging		
	Jung et al.	2	2 (100)
	***Total***	***337***	***59*** ± ***18***
	Standard OCTA Imaging		
*Non-flow area DCP*	Pahl et al.	30	6 (20)
	Falavarjani et al.	18	10 (55)
	Minvielle et al.	18	5 (3)
	Sambhav et al.	3	3 (100)
	Grover et al.	1	1 (100)
	Martin et al.	6	6 (100)
	Mgboji et al.	4	4 (100)
	Mokrane et al.	46	10 (22)
	Han et al. [33]	82	16 (38)
	Pinhas et al.	11	3 (27)
	Han et al. [19]	10	3 (30)
	Sanfilippo et al.	2	1 (50)
	Ong et al.	68	27 (40)
	Abdelkader et al.	14	10 (71)
	Grego et al.	36	13 (36)
	Widefield OCTA Imaging		
	Jung et al.	2	2 (100)
	***Total***	***351***	***60*** ± ***20***
*FAZ Irregularity*	Standard OCTA Imaging		
	Roemer et al.	11	9 (81)
	Falavarjani et al.	18	6 (33)
	Minvielle et al.	18	15 (83)
	Mgboji et al.	4	2 (50)
	Pinhas et al.	11	3 (27)
	***Total***	***62***	***54*** ± ***13***
*FAZ Enlargement*	Standard OCTA Imaging		
	Minvielle et al.	18	18 (100)
	Mgboji et al.	4	2 (50)
	Pinhas et al.	11	2 (18)
	Han et al. [19]	10	1 (10)
	***Total***	***143***	***45*** ± ***20***
*Vascular Tortuosity*	Standard OCTA Imaging		
	Pahl et al.	32	10 (31)
*Vascular Enlargement*	Standard OCTA Imaging		
	Mokrane et al.	46	36 (78)
*Disruption of the perifoveal anastomotic capillary arcade n (%)*	Standard OCTA Imaging		
	Mokrane et al.	46	29 (63)

### Quantitative features of sickle cell disease on standard OCTA imaging

**Fig. 2:**
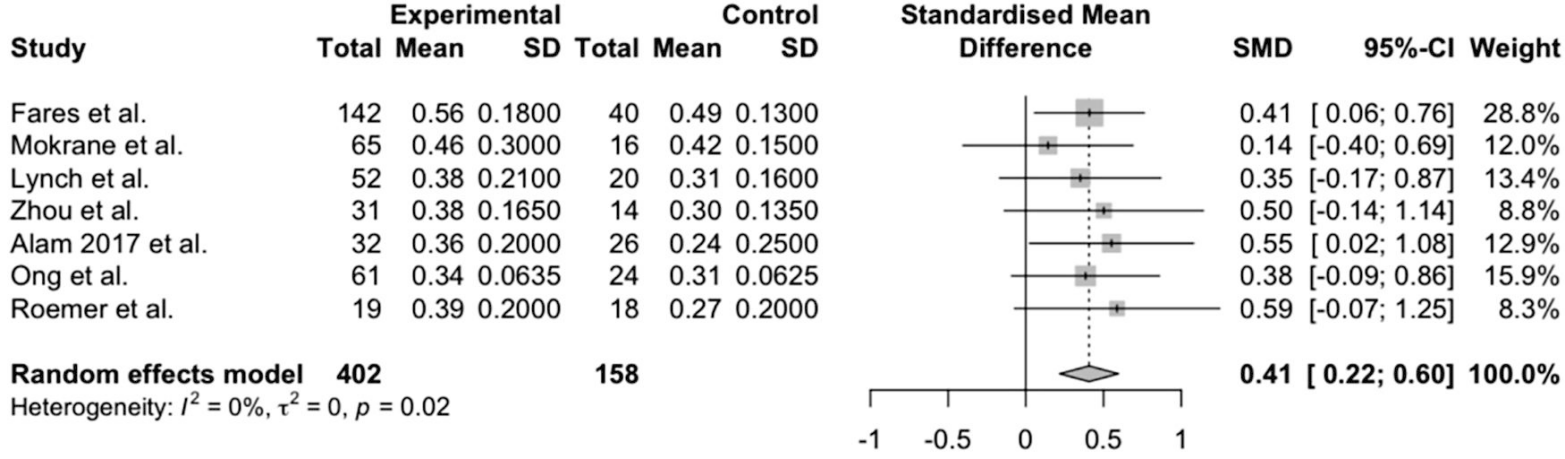
A meta-analysis of seven studies reporting FAZ area in SCR compared to healthy controls. The mean FAZ area was significantly larger in patients with SCR than in healthy controls.

**Fig. 3:**
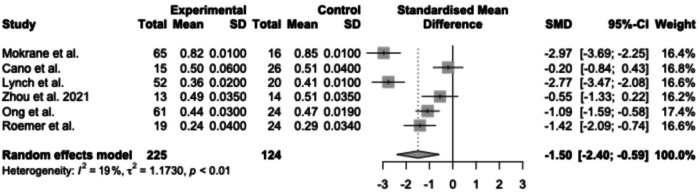
A meta-analysis of six studies reporting mean parafoveal vessel density (SCP) in SCR compared to healthy controls. Vessel density was significantly lower in patients with SCR than in healthy controls.

Many studies presented their OCTA imaging results as continuous variables, including FAZ area, vascular density, tortuosity index, space between small vessels, space between large vessels, total retinal blood flow and ischaemic index. A forest plot displaying results from a meta-analysis of seven studies reporting FAZ area in SCR compared to healthy controls is shown in Fig. 2 [8, 11, 17, 21, 29, 34, 37]. The mean FAZ area was significantly larger in patients with SCR than in healthy controls that were unmatched in one study [21] and matched for age [11, 17, 29, 34, 37], race [8, 11, 17, 29, 34, 37], gender [11] and axial length [34] in the remaining studies. A forest plot displaying a meta-analysis of a further seven studies reporting mean parafoveal SCP and DCP vessel density in SCR compared to healthy controls is shown in Fig. 3. Mean vessel density was significantly lower in patients with SCR than healthy controls. [11, 17, 28, 29, 34, 37].

Following meta-analysis, visual assessment for publication bias using funnel plots showed equal distribution of the studies’ effect sizes above and below the overall line of effect size estimate. Eggers regression coefficient was not performed as a sufficient number of studies were not included in the analysis.

Several studies reported quantitative OCTA outcomes, which were not suitable for meta-analysis. Zhou et al. have used two episodes of serial OCTA scans taken a short time interval apart to show the dynamic variation of capillary perfusion in patients with SCR compared to controls. Alam et al. measured the sensitivity of OCTA parameters against traditional retinal thickness analysis for SCR detection [11]. The most sensitive parameters were the contour irregularity of FAZ in the superficial layer and avascular density in temporal regions, while the area of FAZ, tortuosity, and mean diameter of the vessel were moderately sensitive. The same research group reported that differential artery-vein analysis can significantly improve the performance of OCTA detection and classification of SCR [22]. Lastly, Khansari et al. used OCTA image processing to generate a quantitative vessel tortuosity index (VTI) based on a combination of local and global centreline features. Vessel tortuosity was higher in SCR than in control subjects in the perifoveal and parafoveal regions (*P* ≤ 0.001) [23].

### Widefield and ultra-widefield OCTA imaging

Two studies included in this review used widefield OCTA imaging (12 × 12 area scans) and therefore, their results cannot be meaningfully contrasted with those from the other studies. Bistour et al. assessed the capillary non-perfusion in different concentric sectors on widefield OCTA and correlated non-perfusion to the severity of SCR [20]. They determined that a 30–80 degree field of view had the best sensitivity and specificity (41.67% and 93.33%, respectively) for distinguishing no SCR from non-proliferative SCR. Jung et al. demonstrated that swept-source OCTA 12 × 12-mm imaging effectively captured a reduction in SCP and DCP densities in the temporal macular region, correlating with areas of retinal thinning. Furthermore, the application of widefield imaging revealed a notable reduction in capillary density in the temporal region of these patients. A case report published by Sanfilippo et al. reported the results of ultra-widefield OCTA in a patient with SCD who was visually asymptomatic. The ultra-widefield OCTA detected reduced flow in the DCP and SCP, the DCP being most affected.

### OCTA imaging and fluorescein fundus angiography (FFA)

Three studies compared imaging results from OCTA and FFA which is the current gold standard for SCR imaging. Minvielle et al. reported that out of 18 patients with SCD, OCTA demonstrated parafoveal abnormalities indicative of maculopathy in all cases, whereas FFA detected abnormality in 50% (9/18). Similarly, Sambhav et al. reported a case series of 3 patients with SCD, all which had normal FFAs but evidence of maculopathy on OCTA and SD-OCT. A case reported by Sanfilippo et al. confirmed this result using ultra-widefield OCTA, demonstrating flow voids primarily in the DCP that did not appear as extensive when conventional imaging protocols using FFA were used.

### Association of OCTA features and genotype

Table 3 summarises the population characteristics of all included studies. Of the 523 participants in the studies that described the genotype, 343 (65.58%) had SS genotype, 104 (19.89%) had SC genotype, 31 (5.93%) had S-thalassaemia genotype, and 31 (9.75%) carried the sickle cell trait. Four studies reported no difference in ischaemic abnormalities detectable via qualitative or quantitative OCTA analysis and patient genotype [6, 16, 17, 34]. However, multivariate regression analyses conducted by Fares et al. showed HbSS genotype was predictive of sickle cell maculopathy (*p* = 0.01) [8]. Similarly, Zhou et al. identified that intermittent perfusion detectable on OCTA was more common in HbSS rather than HbSC genotypes [11]. Only one case series detected a greater occurrence of ischaemic changes in OCTA subfields for patients with SC genotype over SS or b-thalassaemia genotypes [19]

**Table 3:** Summary of participant characteristics.

Reference	Sample (eyes)	Control sample (eyes)	Mean age (sample)	Mean age (control)	Sex (%)		Genotype (%)				Goldberg Classification (% eyes)							Retinopathy Classification (% eyes)		
					M	F	SS	SC	S Thal	HbS variant	AS	0	1	2	3	4	5	NP	P	no SCR
Mgboji et al.	2 (4)	–	32	–	1 (50)	1 (50)	1 (50)	1 (50)	–	–	2	0	0	0	0	2	0	2	2	2
Minvielle et al.	9 (18)	5 (9)	41	38	5 (56)	4 (44)	8 (44)	10 (51)	1 (5)	–	0	0	6	8	4	0	0	14	4	0
Pahl et al.	16 (32)	5 (10)	15	17	–	–	10 (63)	5 (37)	–	–	0	0	4	20	0	0	0	24	0	0
Fares et al.	78 (151)	20 (40)	37	–	47 (32)	53 (68)	45 (58)	–	–	33 (42)	–	–	-	-	-	-	-	-	-	-
Mokrane et al.	24 (46)	8 (16)	34	37.5	8 (33)	16 (66)	14 (58)	10 (42)	–	–	–	–	-	-	-	-	-	-	-	-
Han et al. [33]	46 (82)	–	36	–	18	28	27 (60)	14 (31)	4 (9)	–	0	–	22	20	18	2	2	14	18	18
Cano et al.	12 (24)	19 (26)	40	36	2 (17)	10 (83)	6 (50)	4 (33)	2 (17)	–	–	–	-	-	-	-	-	7	10	-
Croisé et al.	31 (56)	–	31	–	20 (65)	11 (35)	20 (64)	7 (23)	4 (13)	–	0	16	12	19	17	0	0	31	17	16
Falavarjani et al.	10 (18)	–	37	–	7 (70)	3 (30)	6 (60)	3 (30)	1 (10)	–	0	0	-	-	-	-	-	12	6	-
Lynch et al.	72 (72)	20 (20)	33	33	26 (36)	46 (74)	35 (67)	10 (19)	7 (14)	–	–	–	-	-	-	-	-	33	19	-
Zhou et al.	31 (62)	14 (28)	34	32	2 (7)	29 (93)	23 (74)	8 (26)	–	–	–	–	-	-	-	-	-	21	10	-
Jung et al.	2 (2)	–	34	–	2 (100)	0	2 (100)	0	0	0	2	0	0	0	0	0	0	0	0	2
Sambhav et al.	3 (3)	–	19	–	2 (66)	1 (33)	3 (100)	0	0	0	–	–	-	-	-	-	-	-	-	-
Martin et al.	3 (6)	–	–	–	3 (100)	0	3 (100)	0	0	0	0	3	0	0	0	0	0	3	0	-
Grover et al.	1 (1)	–	19	–	1 (100)	0	1 (100)	0	0	0	0	1	0	0	0	0	0	1	0	0
Monteiro et al.	16 (32)	–	16	–	9 (56)	7 (44)	16 (100)	–	–	–	–	–	-	-	-	-	-	1	2	13
Alam et al.	10 (19)	10 (18)	40	20	4	6	10 (100)	0	0	0	0	0	2	5	1	0	0	7	1	-
Khansari et al.	34 (34)	17 (17)	41.5	39	8 (23)	26 (77)	19 (56)	11 (32)	4 (12)	0	0	1	1	22	8	2	0	24	10	-
Alam et al. [22]	48 (85)	20 (40)	42.5	42	28 (58)	20 (42)	–	–	0	0	0	0	15	15	6	10	2	30	18	-
Alam et al. [10]	18 (32)	13 (26)	40	37	7 (29)	11 (71)	–	–	–	–	–	–	-	-	-	-	-	-	-	-
Zhou et al. [11]	13 (13)	14 (14)	31	27	6 (46)	7 (54)	8 (62)	4 (38)	–	–	–	–	-	-	-	-	-	-	-	-
Pinhas et al.	11 (11)	1 (1)	33	34	3 (27)	8 (73)	9 (82)	2 (18)	0	0	0	0	4	4	1	1	1	8	3	-
Bistour et al.	28 (42)	–	–	–	–	–	–	–	–	–	–	–	-	-	-	-	-	14	15	13
Han et al. [6]	19 (36)	–	31	–	9 (47)	10 (53)	26 (72)	8 (22)	2		0	0	4	23	8	1	0	27	9	-
Han et al. [19]	5 (10)	–	37.6	–	1 (33)	2 (66)	3 (100)	0	0	0	–	–	-	-	-	-	-	2	1	-
Sanfilippo et al.	1 (2)	–	33	–	1 (100)	0	1 (100)	0	0	0	1	0	0	0	0	0	0	1	0	1
Ong et al.	34 (68)	12 (24)	17	11	8 (47)	9 (53)	17 (50)	–	–	17 (50)	–	–	5	33	2	-	-	39	2	27
Abdelkader et al.	15 (30)	–	11	–	9 (60)	6 (40)	8 (53)	3 (20)	4 (27)	–	–	–	-	-	-	-	-	30	0	-
Grego et al.	18 (36)	–	11	–	15 (83)	3 (17)	12 (67)	4 (22)	2 (11)	–	–	–	-	-	-	0	0	16	0	23
Roemer et al.	10 (19)	9 (18)	8	11	4 (40)	6 (60)	10 (100)	0	0	0	0	0	2	5	1	0	0	7	1	-

### Association of OCTA features and visual acuity

Sixteen studies recorded visual acuity (53%), with all bar one reporting participants’ average visual acuity as 20/25 or better. Han et al. [33] found visual acuity correlated with FAZ area and parafoveal vascular density in the superficial and deep plexi [33]. No other studies measured the association between visual acuity and OCTA outcomes.

### Association of OCTA features and retinal thinning

**Fig. 4:**
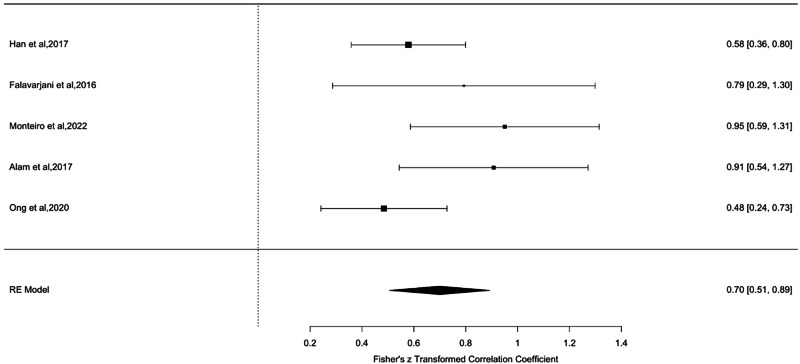
Meta-analysis of the correlation between temporal para-foveal retinal thickness and superficial capillary plexus density. This forest plot summarizes the results of five studies evaluating the correlation between temporal para-foveal retinal thickness and superficial capillary plexus (SCP) density, expressed as Fisher’s z-transformed correlation coefficients. Each study is represented by a square (point estimate), with horizontal lines denoting 95% confidence intervals. The size of each square reflects the study’s weight in the meta-analysis. The overall pooled effect, calculated using a random-effects (RE) model, is shown as a diamond at the bottom of the plot. A significant positive correlation was found, with a pooled Fisher’s z value of 0.70 [95% CI: 0.51, 0.89], indicating that greater para-foveal thickness is associated with increased SCP density.

This review identified five studies assessing the correlation between the temporal para-foveal thickness and superficial capillary plexus density. A forest plot displaying the meta-analysis results is shown in Fig. 4 [1, 10, 17, 30, 33] Insufficient data across all studies was available to correlate other retinal areas and deep capillary plexus findings. One study that attempted to correlate retinal thickness values across superior, nasal, and inferior subfields found that the temporal subfield was significantly thinner in patients with SCR. The temporal subfield was also more likely to yield lower quantitative measures of superficially capillary plexus density than the other subfields [33].

### Association of OCTA features and serological markers of sickle cell disease severity

While OCTA findings were not directly correlated with blood test results in any of the included studies, two studies reported a statistically significant association between the presence of sickle cell maculopathy and Hb, Htc; higher MCV, percentage of reticulocytes, higher levels of reticulocytes and total bilirubin [8, 38]. Grego et al. found a significant association between the presence of sickle cell maculopathy and lower foetal Hb, low prothrombin ratio and increased LDH [8]. Abdelkader et al. reported that longer disease duration, higher reticulocyte %, more painful crises, and noncompliance with hydroxyurea medication were linked to eye abnormalities on fundus examination and OCTA [16]. Two studies used OCTA to show reperfusion of retinal capillaries after hydroxyurea therapy [10, 12, 21].

### Association of OCTA features and Goldberg staging

The classification of retinopathy, as proposed by Goldberg et al., was described in 26 articles. Of the 564 eyes included in the studies that described the degree of SCR, 59 (10.5%) showed no signs of retinopathy, 442 (59.0%) had non-proliferative retinopathy, and 144 (25.53%) had proliferative retinopathy (Goldberg Stages 11-V). Correlating Goldberg stage and OCTA outputs is useful to understand the relationship between sickle cell maculopathy and peripheral retinal changes in SCR. However only two studies analysed the relationship between these data points. One study found no statistical difference in terms of FAZ area or vascular density by stage of Goldberg classification [34]. However, a retrospective case series conducted by Croise et al. reported a significant negative correlation between the mean and temporal density of the superficial and deep plexus capillaries (*p* = 0.0009) and the Goldberg stage on fluorescein angiography [31]. Hence, the relationship between macular changes and peripheral retinal disease in patients with sickle cell disease remains inconclusive.

## Discussion

This systematic review and meta-analysis aimed to rigorously search the literature, providing an analysis of the current understanding of the role of OCTA in SCR diagnosis and monitoring. A total of 31 studies met our inclusion criteria [1, 6, 8–12, 16–36], with 17 (57%) of these studies being published since a previous systematic review was conducted by Guerra et al. in 2019 [13]. Significantly, the growing evidence base exploring OCTA as a tool for diagnosis and staging of SCR enabled us to conduct the first quantitative synthesis of published data, identifying several statistically significant markers of macular ischaemia in patients with SCR. A discussion of the review’s narrative and quantitative findings contextualised within the existing literature is detailed below.

The meta-analysis results show that OCTA is a useful tool for isolating quantitative markers of SCR. Across the studies in this review, FAZ size and vessel density were the most widely reported OCTA metrics. Mean vessel density was significantly lower in patients with SCR than in healthy controls [11, 17, 28, 29, 34, 37]. The FAZ area was larger, indicating that both could represent useful markers of sub-clinical macular ischaemia in patients with SCR [8, 11, 17, 21, 29, 34, 37]. Notably, several studies achieved greater sensitivity and specificity for SCR detection by combining several OCTA parameters to generate a single metric that indicates macular ischaemia. For example, Alam et al. conducted computer-aided SCR classification using six quantitative OCTA parameters, highlighting the potential for OCTA to produce biomarkers that facilitate objective and automated SCR classification [10]. Developments in this area could be instrumental in setting up cost-effective screening programmes for SCR utilising OCTA as a rapid, non-invasive primary screening tool.

Additionally, less commonly reported quantitative indicators of retinovascular remodelling, such as vessel tortuosity [23, 35], space between vessels [32] and fractal dimension [21], were found to be of value in detecting macular ischaemia in patients with SCR. However, analysis of less widely utilised OCTA measures requires specialist expertise in image processing and analysis, and therefore, despite the widespread use of OCTA, its potential to shed light on the vascular remodelling taking place in SCR remains relatively untapped. However, built-in software that automatically provides quantitative metrics is on the horizon and would quickly advance the clinical utility of quantitative OCTA metrics [39].

As anticipated, studies in this review showed a positive correlation between the retinal thinning on SD-OCT ETDRS retinal thickness maps and OCTA assessments of vessel density [1, 10, 17, 30, 33]. Hence, as demonstrated by Grego et al., OCTA has the potential to help characterise the distinct process of vascular remodelling that underlies the characteristic temporal retinal thinning widely documented in SCR [38]. Furthermore, OCTA has been demonstrated to work synergistically with additional imaging techniques, such as adaptive optics scanning light ophthalmoscopy, to improve SCR prognostication and predict response to systemic treatment. Therefore, the potential to characterise the pathophysiology of SCR and identify non-invasive biomarkers of SCR progression utilising a combination of OCTA metrics and multimodal imaging is promising. [30].

Notably, due to their small sample size, some studies in this review opted to report the relative frequencies of qualitative findings, such as the presence of flow voids or FAZ enlargement, rather than conducting a quantitative analysis. Generalisations of qualitative data analysis across studies cannot be made due to inter-study variations in image analysis and the inherent subjectivity of qualitative image grading. However, it is reassuring that where qualitative analysis was performed, results aligned with quantitative findings. FAZ size and flow voids in the DCP and SCP were assessed by most graders to be increased in patients with SCR, suggesting clinicians with limited expertise in OCTA data analysis may be able to utilise OCTA clinically to aid diagnosis and clinical decision-making (Table 2).

Importantly, several studies contrasted the role of OCTA with that of the current gold standard, FFA [5]. OCTA has garnered attention due to its non-invasive nature and ease of use [26], and studies in this review highlight the increased sensitivity of OCTA in detecting macular microvascular abnormalities in SCR compared to FFA [9, 26]. Minvielle et al. demonstrated that OCTA identified parafoveal vascular changes in all patients with SCD, whereas FFA detected abnormalities in only half [9]. Similarly, the case series by Sambhav et al. found that all patients exhibited normal FFA findings despite evidence of macular changes on OCTA [26]. This discrepancy is likely due to OCTA’s ability to provide depth-resolved imaging of the retinal capillary plexuses, particularly the DCP, which appears disproportionately affected in SCR [9, 26].

Furthermore, Sanfilippo et al. demonstrated that ultra-widefield OCTA can reveal DCP flow deficits that are less apparent on conventional FFA, suggesting its potential for comprehensive assessment of microvascular compromise extending into the retinal far periphery, an area of premium importance in SCR [18]. Nonetheless, while ultra-widefield OCTA may help bridge the gap between OCTA and FFA [18], FFA remains the gold standard due to its ability to reliably detect hallmark SCR features, including peripheral ischaemia, vascular leakage, arteriovenous anastomoses, and sea-fan neovascularization [5]. Further research is needed to establish the clinical utility of ultra-widefield OCTA and determine its optimal integration alongside FFA for the diagnosis and monitoring of SCR.

Concerning participant characteristics, it is significant that despite many of the included studies documenting participant data such as Goldberg stage, genotype, serology, and visual acuity, only a few studies had sufficient power to establish correlations between these metrics and OCTA changes. Consequently, our ability to establish associations between patient characteristics and OCTA imaging is limited. The absence of analytical statistics examining the relationship between the Goldberg stage—a grading system emphasizing peripheral retinal changes—and OCTA scans centred on the fovea is a significant limitation. This gap hinders our understanding of whether peripheral and central disease processes are distinct entities or represent different stages of a unified pathophysiological mechanism. Moreover, there is a pressing need for a more comprehensive understanding of the risk factors predicting adverse outcomes in SCR, given the non-linear evolution of its disease course and striking phenotypic diversity exhibited by each genotype [40]. For example, some patients with SCR progress rapidly, developing sight threatening complications, while others appear to be safeguarded by the phenomenon of autoinfarction, wherein bleeding vessels spontaneously occlude [41]. Moreover, investigating which imaging OCTA biomarkers are associated with adverse clinical outcomes (such as reduced visual acuity) and how these relate to existing feature-based grading systems is critical for determining the clinical utility of OCTA.

Patient genotype was measured by 27 out of 30 studies (90%). The HbSC genotype is commonly thought to possess the highest risk of proliferative SCR development [39], while HbSS is associated with non-proliferative SCR. However, in this review, only one cross-sectional study conducted by Han et al., with a relatively small sample size of ten eyes, corroborated this finding [19]. Instead, most studies showed no relationship between genotype and SCR. Surprisingly, the cross-sectional analysis conducted by Fares et al., which utilised a dataset of 151 eyes, reported that HbSS genotype, rather than HbSC genotype, was associated with an increased risk of sickle cell maculopathy [8]. It has been posited that genetic differences in endothelial function in patients with sickle cell disease may contribute to its phenotypic diversity. Endothelial cells that release VEGF in response to inflammation, particularly when unopposed by growth-inhibiting factors like Thrombospondin-A, are more likely to develop neovascular SCR. However, accounting for diverse endothelial responses and, therefore, phenotypic variability risk stratifying genotypes presents a challenge and may explain the inconsistent findings in this review [42]. Furthermore, it is important to highlight that out of all participants included in the studies synthesised within this review, only 19.89% of participants were of the HbSC genotype. While this reflects the population prevalence of HbSS and HbSC genotypes, future research should focus on sampling those with HbSC genotypes, such that sample sizes of HbSC patients suitable for analytical statistics can be generated.

Additionally, serological markers for disease activity could prove pivotal in enhancing our understanding of which patients with sickle cell disease are most likely to develop active SCR. Two cross-sectional studies linked lower levels of foetal Hb, high reticulocyte count and prothrombin ratio to evidence of macular ischaemia on OCTA, indicating that patients with hypercoagulability may be less at risk of retinal complications [8, 38]. While this finding aligns with the hypothesis that hypercoagulability may promote autoinfarction and be protective in the retina, more research is required to prove the link between serological markers and SCR progression in patients with sickle cell disease.

Understanding the relationship between OCTA metrics and clinical outcomes, such as visual acuity, is key to determining the clinical utility of OCTA imaging. While many studies recorded participant visual acuity, reporting a high mean visual acuity of 20/20 across study participants, only Han et al. correlated visual acuity data with OCTA findings [33]. Han et al. recorded the visual acuity of 46 patients with SCR and found a significantly positive correlation between the extent of parafoveal flow loss (assessed qualitatively) and patients’ logMar acuity [33]. This finding complements previous studies that have successfully correlated OCTA quantitative measures such as FAZ size and macular vascular density with visual acuity in diabetic retinopathy and vein occlusions [43]. However, based on this study alone, no conclusions can be drawn about which OCTA changes are likely to be subclinical and which could lead to a fall in acuity. Altogether, more evidence is needed to characterise the relationship between OCTA markers of vascular remodelling and visual outcomes.

The Goldberg staging system, produced in 1980, remains the most widely recognised system for grading the severity of SCR [3]. The feature-based stages use macroscopic retinal changes to predict which patients are most likely to develop clinically significant, sight-threatening complications. Only one study in this review that attempted to correlate OCTA imaging findings and Goldberg staging could prove a statistically significant relationship [31]. The lack of correlation may indicate two distinct disease processes, i.e. macular disease and peripheral disease in people with SCD. However, statistically significant correlations between markers of macular ischaemia identified on OCTA and evidence of temporal retinal thinning on SD OCT increasingly suggest macular and peripheral remodelling are interrelated [6]. Alternatively, the lack of correlation may reflect the limited sample sizes in many of the studies included in this review. A grading system for SCR, which incorporates microscopic indicators of vascular remodelling in the macula, could aid SCR staging and prognostication.

### Limitations and future research

A key strength of this review was the quantitative analysis of several OCTA metrics across several studies evaluating SCR in patients with sickle cell disease. Nonetheless, the conclusions of our analysis remain dependent on the quality of the available evidence. All studies that met our inclusion criteria were observational rather than randomised controlled trials. Consequently, uncontrolled confounders limit the extent to which conclusions can be drawn about cause and effect. The heterogeneity in OCTA technology, imaging schedules, and reporting between studies also limited our findings and meant some evidence could not be included in our final analysis. For example, only studies with comparable imaging areas could be combined and it is possible that incorporating Heidelberg, Optovue and Topcan devices introduces bias. Moreover, the small sample size available for evaluation included in most studies made it difficult to draw statistically significant associations between participant characteristics and OCTA imaging results. Additionally, the studies included in this review sought to identify statistically significant differences between patients with SCR and healthy controls. However, it is well established that OCTA metrics, such as FAZ size, exhibit considerable variation within healthy populations due to confounding factors such as age and sex (https://www.sciencedirect.com/science/article/abs/pii/S0014483519305895). Consequently, these findings must be interpreted within the context of population-level data, emphasizing the need for further studies to establish normative OCTA parameters across diverse age groups, genders, and ethnicities.

Future research focusing on prospective, longitudinal analysis of OCTA data from a large cohort of patients with SCR would be of great value in consolidating our understanding of how OCTA could be used to improve SCR diagnosis and management. The movement toward developing national and international imaging banks similar to those available for diabetic eye disease would be of great value in linking imaging results with clinical outcomes such as visual acuity or sight-threatening complications. The aggregation of such large imaging datasets, combined with advancements in machine learning, could aid the development of automated SCR screening and detection [44]. Furthermore, an improved understanding of which patients are at risk of ocular and systemic disease progression based on OCTA imaging data could prove vital for guiding the management of both ocular and systemic treatments for patients with sickle cell disease.

## Conclusion

OCTA is a widely available, non-invasive, and sensitive tool for quantitatively analysing vascular remodelling in the macula of patients with SCR, offering advantages over FFA in imaging the layered macular vasculature. However, FFA remains the gold standard for SCR imaging due to its ability to comprehensively assess peripheral ischaemia, vascular leakage, arteriovenous anastomoses, and sea-fan neovascularization. While conventional OCTA is limited to the posterior pole, the development of widefield OCTA has extended its reach to the peripheral retina, potentially narrowing the gap between OCTA and FFA. Nonetheless, challenges such as motion artifacts, projection artifacts, and limited field of view compared to FFA remain. Current data interpretation is further constrained by small sample sizes, heterogeneity of published data, and a scarcity of evidence linking imaging findings to clinical outcomes. Establishing an imaging database may improve our understanding of vascular remodelling in SCR and support the development of automated algorithms for detecting retinal biomarkers predictive of disease progression. Further studies are essential to validate these imaging approaches and define their role in clinical practice.

## Supplementary information

STROBE Checklist

Bias assessment results table (GRADE)
