# Supplementary material for: Optical coherence tomography angiography as a tool for diagnosis and monitoring of sickle cell related eye disease: a systematic review and meta-analysis
Source: Eye (Lond). 2025 May 22;39(11):2112–23. doi: 10.1038/s41433-025-03814-1 (PMC12274534; doi:10.1038/s41433-025-03814-1)
Supplement: Supplementary file 1 — STROBE Checklist [file 41433_2025_3814_MOESM1_ESM.pdf]

Item		Item	STROBE Guidelines	Mgboji et al.	Minvielle et al.	Pahl et al.	Fares et al.	Mokrane et l.	Han et al. 2017	Cano et al.	Croisé et al.	Falavarjani et al.	Lynch et al.	Zhou et al.	Jung et al.	Sambhav et al.	Martin et al.	Grover et al.	Monteiro et al.	Alamet al.	Khansari et al.	Alam2019 et al.	Alam2017 et al.	Zhou 2021,	Pinhas et al.	Bistour et al	Han2018	Han2015	Sanfilippo-	Ong et al.t	Abdelkader et al	Grego et al	Romer et al.
Title and abstract		1	(a) Indicate the study's design with a commonly used term in the title or the abstract	N	Y	Y	Y	Y	Y	N	Y	Y	Y	Y	Y	Y	Y	Y	N	N	N	Y	N	N	Y	Y	Y	N	Y	Y	Y	N	Y
			(b) Provide in the abstract an informative and balanced summary of what was done and what was found	Y	Y	Y	Y	Y	Y	Y	Y	Y	Y	Y	Y	Y	Y	Y	Y	Y	Y	Y	N	Y	Y	Y	Y	Y	Y	Y	Y	Y	Y
Introduction																																	
	Background/rationale	2	Explain the scientific background and rationale for the investigation being reported	Y	Y	Y	Y	Y	Y	Y	Y	Y	Y	Y	Y	Y	Y	Y	Y	Y	Y	Y	Y	Y	Y	Y	Y	Y	Y	Y	Y	Y	Y
	Objectives	3	State-specific objectives, including any prespecified hypotheses	Y	Y	Y	Y	Y	Y	Y	Y	Y	Y	Y	Y	Y	Y	Y	Y	Y	Y	Y	Y	Y	Y	Y	Y	Y	N/A	Y	Y	Y	Y
Methods																																	
	Study design	4	Present key elements of study design early in the paper	Y	Y	Y	Y	Y	Y	Y	Y	Y	Y	Y	Y	Y	Y	Y	N	N	N	Y	N	N	Y	Y	Y	Y	Y	Y	Y	N	Y
	Setting	5	Describe the setting, locations, and relevant dates, including periods of recruitment, exposure, follow-up, and data collection	Y	Y	Y	Y	Y	N	Y	Y	Y	Y	Y	Y	Y	Y	Y	Y	Y	Y	Y	N	Y	Y	Y	N	Y	Y	Y	Y	Y	Y

	Participants	6	(a) Cohort study—Give the eligibility criteria and the sources and methods of selection of participants. Describe methods of follow upCase-control study—Give the eligibility criteria, and the sources and methods of case ascertainment and control selection. Give the rationale for the choice of cases and controlsCross-sectional study—Give the eligibility criteria, and the sources and methods of selection of participants	N/A	Y	Y	Y	Y	Y	N	Y	Y	Y	Y	Y	Y	Y	N/A	Y	Y	Y	N	N	Y	Y	Y	Y	N	N/A	Y	N	Y	Y
			(b) Cohort study—For matched studies, give matching criteria and number of exposed and unexposedCase-control study—For matched studies, give matching criteria and the number of controls per case	N/A	Y	Y	Y	Y	Y	Y	N	Y	Y	Y	N/A	N/A	N/A	N/A	Y	Y	Y	Y	Y	Y	N	N/A	Y	N	N/A	Y	Y	N	Y
	Variables	7	Clearly define all outcomes, exposures, predictors, potential confounders, and effect modifiers. Give diagnostic criteria, if applicable	Y	Y	Y	Y	Y	Y	Y	Y	Y	Y	Y	Y	Y	Y	Y	Y	Y	Y	Y	Y	Y	Y	Y	Y	Y	N/A	Y	Y	Y	Y
	Data sources/measurement	8	For each variable of interest, give sources of data and details of methods of assessment (measurement). Describe comparability of assessment methods if there is more than one group	Y	Y	Y	Y	Y	Y	Y	Y	Y	Y	Y	Y	Y	Y	Y	Y	Y	Y	Y	Y	Y	Y	Y	Y	Y	Y	Y	Y	Y	Y
	Bias	9	Describe any efforts to address potential sources of bias	N	Y	Y	Y	Y	N	N	N	Y	N	Y	N/A	N/A	N/A	N/A	Y	N	Y	Y	N	N	Y	Y	Y	N	N/A	Y	N	N	N
	Study size	10	Explain how the study size was arrived at	N	N	N	N	N	N	N	N	N	N	N/A	N/A	N/A	N/A	N/A	Y	Y	Y	N	N	Y	N	N	N	N	N/A	Y	N	N	Y
	Quantitative variables	11	Explain how quantitative variables were handled in the analyses. If applicable, describe which groupings were chosen, and why	Y	Y	Y	Y	Y	Y	Y	Y	Y	Y	Y	N/A	N/A	N/A	N/A	Y	Y	Y	Y	Y	Y	Y	Y	Y	N	Y	Y	Y	Y	Y
	Statistical methods	12	(a) Describe all statistical methods, including those used to control for confounding	N	Y	N	Y	Y	Y	Y	Y	Y	Y	Y	N/A	N/A	N/A	N/A	Y	Y	Y	Y	Y	Y	Y	Y	Y	N	N/A	Y	Y	Y	Y
			(b) Describe any methods used to examine subgroups and interactions	Y	Y	N	N	Y	Y	Y	Y	Y	Y	Y	N/A	N/A	N/A	N/A	Y	Y	Y	Y	Y	Y	Y	Y	Y	N	N/A	Y	Y	Y	Y

			(c) Explain how missing data were addressed	N/A	Y	N	Y	N	Y	Y	N	Y	Y	Y	N/A	N/A	N/A	N/A	N	Y	N	Y	Y	Y	Y	Y	Y	N	N/A	Y	Y	Y	N
			(d) Cohort study—If applicable, explain how loss to follow up was addressedCase-control study—If applicable, explain how matching of cases and controls was addressedCross-sectional study—If applicable, describe analytical methods taking account of sampling strategy	N/A	Y	N	Y	N	Y	Y	Y	Y	Y	N/A	N/A	N/A	N/A	N/A	Y	Y	Y	N/A	Y	Y	Y	N	Y	N	N/A	Y	Y	N/A	N/A
			(e) Describe any sensitivity analyses	N	Y	N	Y	Y	Y	Y	Y	Y	Y	Y	N/A	N/A	N/A	N/A	Y	Y	Y	Y	Y	Y	Y	Y	Y	N	N/A	Y	Y	Y	Y
Results																																	
	Participants	13	(a) Report the numbers of individuals at each stage of the study—e.g., numbers potentially eligible, examined for eligibility, confirmed eligible, included in the study, completing follow up, and analyzed	Y	Y	Y	Y	Y	Y	Y	N	Y	Y	Y	N/A	N/A	N/A	N/A	Y	Y	Y	Y	Y	Y	Y	Y	Y	Y	N/A	Y	Y	N	Y
			(b) Give reasons for nonparticipation at each stage	N/A	Y	Y	Y	N	Y	Y	Y	Y	Y	Y	N/A	N/A	N/A	N/A	N	Y	Y	Y	Y	Y	Y	Y	Y	Y	N/A	Y	Y	N	N
			(c) Consider use of a flow diagram	N	N	N	Y	N	N	N	N	N	N	N	N/A	N/A	N/A	N/A	N	N	N	N	N	N	N	N	N	N	N/A	N	Y	N	N
	Descriptive data	14	(a) Give characteristics of study participants (e.g., demographic, clinical, social) and information on exposures and potential confounders	Y	Y	Y	Y	Y	Y	Y	Y	Y	Y	Y	N/A	N/A	N/A	Y	Y	Y	Y	Y	N	Y	Y	N	Y	Y	N/A	Y	Y	Y	Y
			(b) Indicate the number of participants with missing data for each variable of interest	N/A	Y	Y	Y	Y	Y	Y	Y	Y	Y	N	N/A	N/A	N/A	N/A	Y	Y	Y	Y	N	Y	Y	Y	Y	Y	N/A	Y	Y	N	Y
			(c) Cohort study—Summarize follow-up time (e.g., average and total amount)	N/A	Y	Y	N/A	N	Y	Y	N/A	Y	N/A	N/A	N/A	N/A	N/A	N/A	Y	Y	N	N/A	N	N/A	N/A	N/A	Y	Y	N/A	Y	N/A	N/A	N/A
	Outcome data	15	Cohort study—Report numbers of outcome events or summary measures over timeCase-control study—Report numbers in each exposure category, or summary measures	Y	Y	Y	Y	Y	Y	Y	N/A	Y	Y	Y	N/A	N/A	N/A	N/A	Y	Y	Y	Y	Y	Y	Y	Y	Y	Y	N/A	Y	Y	Y	Y

			of exposure across-sectional study—Report numbers of outcome events or summary measures																														
	Main results	16	(a) Give unadjusted estimates and, if applicable, confounder-adjusted estimates and their precision (e.g., 95% confidence interval). Make clear which confounders were adjusted for and why they were included	N	Y	Y	Y	Y	Y	Y	Y	Y	Y	Y	N/A	N/A	N/A	N/A	Y	Y	Y	Y	Y	Y	N	Y	Y	Y	N/A	Y	Y	Y	Y
			(b) Report category boundaries when continuous variables were categorised	N	Y	Y	N	Y	Y	Y	Y	Y	Y	Y	N/A	N/A	N/A	N/A	Y	Y	Y	Y	Y	Y	Y	Y	Y	Y	N/A	Y	Y	Y	Y
			(c) If relevant, consider translating estimates of relative risk into absolute risk for a meaningful time period	N/A	Y	Y	N/A	N	Y	N/A	Y	Y	N/A	N/A	N/A	N/A	N/A	N/A	Y	Y	Y	Y	Y	Y	N/A	N/A	Y	Y	N/A	Y	Y	N/A	Y
	Other analyses	17	Report other analyses done—e.g., analyses of subgroups and interactions and sensitivity analyses	N	Y	Y	Y	Y	Y	Y	Y	Y	Y	Y	N/A	N/A	N/A	N/A	Y	Y	Y	Y	Y	Y	N	Y	Y	Y	N/A	Y	Y	Y	Y
Discussion																																	
	Key results	18	Summarise key results with reference to study objectives	Y	Y	Y	Y	Y	Y	Y	Y	Y	Y	Y	Y	Y	Y	Y	Y	Y	Y	Y	Y	Y	Y	Y	Y	Y	Y	Y	Y	Y	Y
	Limitations	19	Discuss the limitations of the study, taking into account sources of potential bias or imprecision. Discuss both the direction and magnitude of any potential bias	N	Y	Y	Y	Y	Y	Y	Y	Y	Y	Y	Y	Y	Y	Y	Y	Y	Y	Y	Y	Y	Y	Y	Y	Y	Y	Y	Y	Y	Y
	Interpretation	20	Give a cautious overall interpretation of results considering objectives, limitations, multiplicity of analyses, results from similar studies, and other relevant evidence	Y	Y	Y	Y	Y	Y	Y	Y	Y	Y	Y	Y	Y	Y	Y	Y	Y	Y	Y	Y	Y	Y	Y	Y	Y	Y	Y	Y	Y	Y
	Generalizability	21	Discuss the generalizability (external validity) of the study results	N	Y	Y	N	N	Y	N	Y	Y	Y	N	N	N	Y	Y	Y	Y	Y	N	Y	N	N	N	Y	Y	Y	Y	N	N	N
Other																																	
	Funding	22	Give the source of funding and the role of the funders for the present study and, if applicable, for the original study on which the present article is based	Y	N	Y	Y	N	Y	Y	Y	N	Y	Y	N	N	Y	Y	Y	Y	Y	Y	Y	Y	Y	Y	Y	Y	N	Y	N	N	N
