# Supplementary material for: Optical coherence tomography angiography as a tool for diagnosis and monitoring of sickle cell related eye disease: a systematic review and meta-analysis
Source: Eye (Lond). 2025 May 22;39(11):2112–23. doi: 10.1038/s41433-025-03814-1 (PMC12274534; doi:10.1038/s41433-025-03814-1)
Supplement: Supplementary file 2 — Bias assessment results table (GRADE) [file 41433_2025_3814_MOESM2_ESM.pdf]

	Risk of Bias	Inconsistency	Indirectness	Imprecision	Publication Bias
Mgboji et al.	🔴	🔴	🟠	🔴	🟠
Minvielle et al.	🔴	🔴	🟠	🔴	🟠
Pahl et al.	🟠	🟠	🟠	🔴	🟢
Fares et al.	🟠	🟢	🟠	🟢	🟢
Mokrane et al.	🟠	🟢	🟠	🟠	🟢
Han et al. 2017	🟠	🟢	🟠	🟠	🟢
Cano et al.	🟠	🟢	🟢	🟠	🟢
Croisé et al.	🟠	🟠	🟠	🟠	🟢
Falavarjani et al.	🟠	🟢	🟠	🟠	🟢
Lynch et al.	🟠	🟢	🟠	🟠	🟢
Zhou et al.	🟠	🟢	🟠	🟠	🟢
Jung et al.	🔴	🟢	🟠	🔴	🟢
Sambhav et al	🔴	🟢	🟠	🔴	🟢
Martin et al.	🔴	🟢	🟠	🔴	🟢
Grover et al	🔴	🟢	🟠	🔴	🟠
Alam et al.	🟠	🟢	🟠	🟠	🟢
Khansari et al .	🟠	🟢	🟠	🟠	🟢
Alam 2019 et al.	🟠	🟢	🟠	🟠	🟢
Alam 2017 et al.	🟠	🟢	🟠	🟠	🟢
Zhou et al 2021	🟠	🟢	🟠	🟠	🟢
Pinhas et a.	🟠	🟢	🟠	🟠	🟢
Bistour et al.	🟠	🟢	🟠	🟠	🟢

Han 2018./	🟠	🟢	🟠	🟠	🟢
Han 2015.	🟠	🟢	🟠	🟠	🟢
Sanfilippo et al.	🔴	🟢	🟠	🔴	🟠
Ong et al.	🟠	🟢	🟠	🟠	🟢
Abdelkader et al.	🟠	🟢	🟠	🔴	🟢
Grego et al.	🟠	🟢	🟠	🟠	🟢
Roemer et al.	🟠	🟢	🟠	🟠	🟢
